# *Escherichia coli* Adhesion and Biofilm Formation on Polymeric Nanostructured Surfaces

**DOI:** 10.1021/acsomega.3c04747

**Published:** 2023-10-09

**Authors:** Divya Iyer, Eric Laws, Dennis LaJeunesse

**Affiliations:** Department of Nanoscience, Joint School of Nanoscience and Nanoengineering, University of North Carolina Greensboro, 2907 East Lee Street, Greensboro, North Carolina 27455, United States

## Abstract

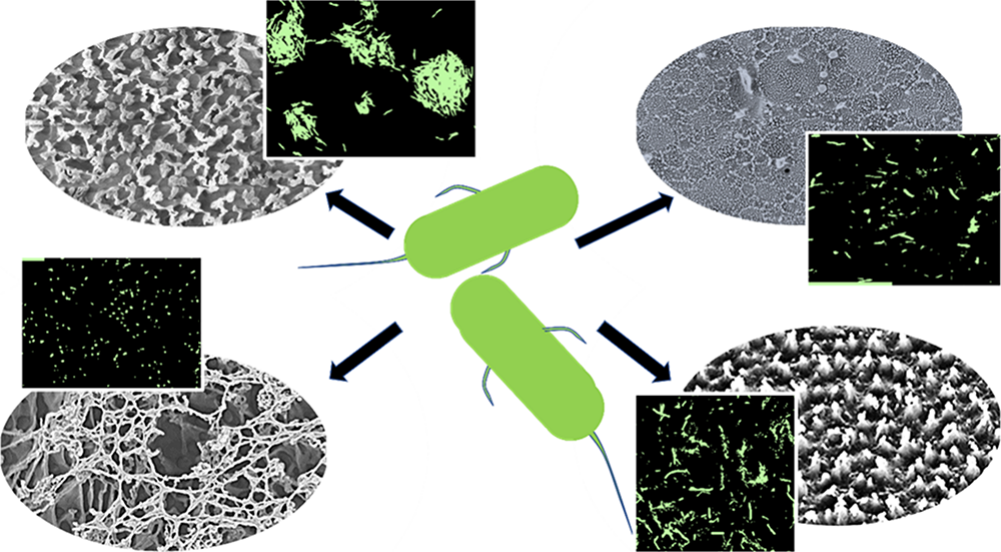

Biofilm formation is a multistep process that requires initial contact between a bacterial cell and a surface substrate. Recent work has shown that nanoscale topologies impact bacterial cell viability; however, less is understood about how nanoscale surface properties impact other aspects of bacterial behavior. In this study, we examine the adhesive, viability, morphology, and colonization behavior of the bacterium *Escherichia coli* on 21 plasma-etched polymeric surfaces. Although we predicted that specific nanoscale surface structures of the surface would control specific aspects of bacterial behavior, we observed no correlation between any bacterial response or surface structures/properties. Instead, it appears that the surface composition of the polymer plays the most significant role in controlling and determining a bacterial response to a substrate, although changes to a polymeric surface via plasma etching alter initial bacteria colonization and morphology.

## Introduction

Biofilms are three-dimensional thin films that are composed of living cells and an array of secreted biological molecules.^1,2^ Interfaces whether between air/liquid or liquid–solid, serve as the ideal locations for biofilm development, and hence, these complex living communities are found on virtually every surface on Earth. Although biofilm composition varies from microbial species to species, they serve similar functions: providing protective barriers from damage, controlling microbial physiology to drive drug resistance, and structurally organized diverse microbial communities that enable adaptation and evolution. Furthermore, the communal aspect of biofilm compounds the risk of antibiotic resistance by creating an environment that promotes the spread of antibiotic-resistant genes through processes like conjugation and transformation.^3^ Microbial biofilm formation is a multistep process that begins with the initial and perhaps most crucial step, the attachment of a planktonic bacterium to a surface.^4,5^ This step is controlled by a variety of factors, and under favorable conditions, adsorbed microbes develop irreversible adhesive interactions which are followed by cellular proliferation, the secretion of extracellular materials, which leads to the formation of a mature biofilm matrix.^2−5^ Surface topology or surface roughness often controls the adsorption/adhesion of a broad range of cell types including microbial cells and has been used widely to improve the adhesion of microbial biofilms in bioreactors.^6,7^ Furthermore, surface properties such as surface energy, surface charge, and surface roughness all play critical roles in bacterial colonization of a surface.^8,9^

In this study, we examine the interaction of *E. coli* bacteria with 21 different polymeric surfaces that have had their surface architecture modified using plasma etching. *Escherichia coli* (*E. coli*) is a model Gram-negative bacterium that has played significant roles in studies of adhesion and biofilm formation.^10^*E. coli* expresses several different modes of adhesion to surfaces through a variety of distinct and well-characterized mechanisms.^11,12^*E. coli* is also responsible for food poisoning and other serious health conditions by contaminating surfaces involved in the food processing industry and agriculture.^13−15^ For these studies, we selected seven polymers that are commonly used in a variety of industrial and biomedical applications and treated these surfaces using plasma dry etching, a common industrial treatment.^16,17^ The intentions of this study are to correlate *E. coli* adhesion phenotypes with specific surface properties. We choose seven polymers for these experiments that have extensive use in agricultural and biomedical applications: polycarbonate (PC), polyimide (PI), perfluoro alkoxy alkane (PFA), polyethylene (PE), acrylonitrile butadiene styrene (ABS), acetal polyoxymethylene (POM), and polyethylene terephthalate (PET). We show that plasma etching of any polymeric surface alters the immediate response of bacterial colonization of a surface, resulting in differences in biofilm deposition, viability, and adhesion. However, these changes are controlled by something other than topography or surface energy and suggest surface composition and not specific surface morphologies or properties.

## Materials and Methods

### Fabrication of Polymeric Nanostructured Surfaces via Reactive Ion Etching

The following polymer substrates were used: polycarbonate (PC), polyimide (PI), perfluoro alkoxy alkane (PFA), polyethylene (PE), acrylonitrile butadiene styrene (ABS), acetal polyoxymethylene (POM), and polyethylene terephthalate (PET; McMaster and Carr). The polymer substrates were thin films and had uniform thickness of 0.005″ (as described in the McMaster and Carr catalogue) /127 μm except for PETG at 0.0625″/1587.5 μm. Polymer thin films were cleaned by ultrasonication for 10 min in isopropyl alcohol (IPA) to remove surface contamination. The samples were etched via oxygen plasma cleaning using a South Bay Technology Model PC-2000 plasma cleaner. Control over the etch directionality was achieved as previously described.^18^ The instrument specifications are as follows: RF discharge at frequency 13.56 MHz capacitively coupled plasma (CCP) operated at forward power 100 W with a chamber pressure 180–200 mT. The exposure times of 10 min for the isotropic etch and 1 min for the anisotropic etch were used. Each polymer sample was cut into 1 cm^2^ squares before use and placed at the bottom of the PEGylated well for assays.

### Scanning Electron Microscopy

All polymeric NSS was characterized using a Zeiss Auriga Scanning Electron Microscope (SEM) located in the shared electron microscopy facility at the Joint School of Nanoscience and Nanoengineering. Images were collected using an accelerating voltage of 5 kV after the deposition of a 5 nm gold/palladium layer using a Leica EM ACE2000 sputter coater. The surfaces containing microbes were prepared as follows: 1 cm^2^ pieces of each polymeric substrate with bacteria were prepared as described above, fixed in Karnovsky’s solution (2.5% glutaraldehyde/2% formaldehyde solution in 1 M cacodylate buffer (pH 7.4)) overnight at 4 °C and dehydrated with an ethanol dehydration series (35%, 50%, 75%, 90%, 95%, 100%). The dehydrated samples were mounted on SEM stubs and sputter coated with 5 nm Au using a Leica EM ACE2000 before SEM analysis at EHT = 3 kV.

### Contact Angle Measurements of NSS Polymeric Surfaces

Static contact angle (CA) measurements were made using the Ramé-Hart 260-F4 contact angle goniometer and analyzed using the DROPimage Advanced software. Two μL of deionized water drops were placed on the surfaces. The contact angles were made on at least 3 different locations on each surface and ten measurements were taken. A standard *t* test, one-way and two way ANOVA tests were performed on all values to determine the statistical difference (*p* < 0.05) in Microsoft Excel.

### Bacterial Strains and Culture

In these experiments, we used *E. coli* BL21 DE3 strain (ECO114, genotype, F– *omp*T *hsdSB* (rB–, mB−) *gal dcm* (DE3) carrying a super folding Green Fluorescent protein variant (GFP) expressing plasmid with an Amp^r^ selection gene (pBad-sfGFP1X, Addgene #51558^19^). For each experiment, a colony was selected from a freshly streaked Luria Broth (LB) plate that contained Ampicillin; cultures were grown overnight in a 5 mL liquid LB medium containing 50ug/mL Ampicillin. All liquid cultures were grown at 37 °C in a shaking incubator. The overnight culture was used to start/spike fresh cultures the next day and adjusted to an OD_600_ of 0.05. The cultures were grown to an OD_600_ of 0.1 measured on a Thermo Scientific NANODROP 2000C. All the assays were performed in PEGylated 24-well plates in a shaking incubator at 37 °C. To induce GFP expression for the detection/counting of individual bacteria, a solution of 20% l-arabinose was added to the *E. coli* culture at a ratio of 1:100 to total culture volume.

### Roughness Calculation

Roughness (Mean Roughness (*S*_a_) and Root Mean Square (*S*_q_) of bulk and etched polymeric surfaces were determined using an Agilent 5600LS AFM.

### Cell Adhesion and Membrane Integrity Assays

The preparation of the microbes for all cellular assay is as follows: 1 mL of an 0.1 OD_600_*E. coli* culture was added to a well in a 24-well Polyethylene Glycol (PEG) treated plate which contained a polymeric sample at the bottom. PEG pretreatment of the well limited the binding of cell to the well bottom and walls, PEG treated was also used as a negative control; the bacteria cells/sample were incubated with the surface for 1 h at 37 °C in a shaking incubator; after incubation, the well/sample was manually washed twice with 1× PBS; then used to perform one of the standard assays: adhesion, membrane integrity/viability, colony unit forming (CFU). For cell adhesion, GFP labeled *E. coli* bacteria were mounted onto a slide and the number of cells/fields of view were manually counted using a Zeiss AxioVision spinning disc confocal microscope. At least 3 images were obtained from each sample at 100×. The total number of cells was counted per field of view and averaged. For membrane integrity, cells were labeled with 0.5 μL/mL acridine orange/propidium iodide in 1XPBS for 1 min followed by another wash with 1× PBS. The fluorescence was assayed using the Zeiss AxioVision spinning disc confocal microscope with ex488/em518 for acridine orange (intact cells) and ex535/em617 Propidium Iodide (permeabilized cells). At least 3 images were obtained from each sample at 100×. The total number of cells in each channel was counted manually and by a Gen5 plate reader and the ratio of red to total cells (red and green labeled cells) was determined and averaged. An additional membrane permeability study was performed using Propidium iodide (PI) to support EtBr observations. All experiments were performed in triplicate with at least 3 biological and 3 technical replicates. A standard *t* test was performed on all values to determine statistical difference (*p* < 0.05) in Microsoft Excel.

## Results

### Fabrication/Characterization of Polymeric NSS Materials

**Figure 1:**
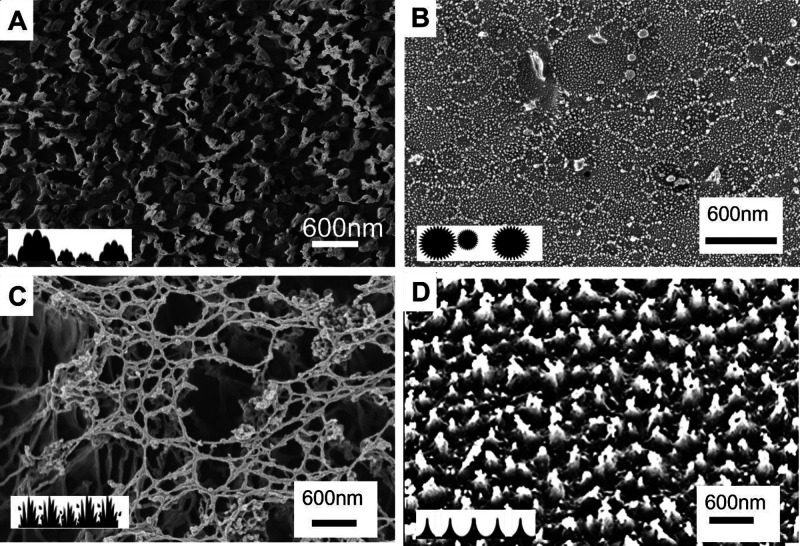
Examples of the nanoscale surface structure of plasma-etched polymeric materials. (A) Anisotropic etched acetal polyoxymethylene (aPOM) exhibiting a “popcorn” surface pattern as depicted in the inset; (B) anisotropic etched polyethylene (aPE) exhibiting a “crater” surface pattern; (C) isotopic etched acetal polyoxymethylene (iPOM) exhibiting a “grass” surface pattern as depicted in the inset; and (D) isotropic etched perfluoroalkoxyalkane (iPFA) exhibiting a “tent” surface pattern as depicted in the inset. All scale bars 600 nm.

To characterize changes in the surface energy of the polymeric surfaces examined in this paper, we performed a static contact angle analysis. Surfaces that demonstrate contact angles below 90° are considered hydrophilic, with contact angles below 10° being superhydrophilic, while those with contact angles greater than 90° are hydrophobic with contact angles over 150° are considered superhydrophobic.^20^ As expected, in most cases, plasma etching of the polymeric surfaces altered the original contact angle (CA) of the bulk material (Table 1; Figure 1; Supplemental Figures 1, 2). CA measurements in our etched nanostructured materials ranging between 8.6° for iPE and the highest angle was iPFA, 132.09 ± 0.04 (Table 1; Supplemental Figure 1, third column, second row). Many etching modifications have been shown to shift the CA, and typically surfaces that are originally hydrophobic in nature become more so (i.e., a higher contact angle) after the generation of nanoscale topology via surface etching, while the opposite is true for surfaces that are originally hydrophilic. We observed a CA shift lower for all but two polymers PFA and POM (Table 1, Supplemental Figure 1, third column; Supplemental Figure 2, second column). In the case of POM, we observed no significant change in CA among all three forms of this material, each having contact angles ranging between 67.1° for the anisotropic etched material and 74.6° and 75.1°, respectively, for the bulk and isotropic etched POM. PFA, which is a hydrophobic material as a bulk material, became more hydrophobic when etched. The remainder of the material became more hydrophilic after etching. The material that showed the greatest change in surface property ABS, which, in its bulk form, had hydrophobic CA of 112.36 ± 0.09°; while isotopically etched ABS was extremely hydrophilic with a CA of 12.1 ± 4.27

**Table 1:** Contact Angle Measurements for Polymeric Surfaces Used in This Study

material	bulk	isotropic etched	anisotropic etched
polycarbonate (PC)	98.6 ± 0.1°	29.7 ± 0.1°	4S.6 ± 0.2°
polyimide (P1)	73.1 ± 0.1°	17.9 ± 0.5°	37.7 ± 0.1°
perfluoroalkoxy alkane (PFA)	111.3 ± 0.2°	132.1 ± 0.1°	121.2 ± 0.1°
polyethylene (PE)	79.0 ± 0.1°	8.6 ± 0.7°	80.5 ± 0.3°
acrylonitrile butadiene styrene (ABS)	112.4 ± 0.1°	12.1 ± 4.3°	60.8 ± 0.2°
acetal polyoxymethylene (POM)	74.6 ± 0.1°	75.1 ± 0.1°	67.3 ± 0.3°
polyethyleneterephhalate (PET)	85.1 ± 0.1°	22.1 ± 0.1°	18.3 ± 0.1°

### Surface Characterization of Polymeric Materials

We characterized all the polymeric surface topology of each sample including bulk, isotropic-etched, and anisotropic-etched using SEM. Plasma-etched polymeric surfaces exhibited four distinct nanoscale topographic configurations: (1) popcorn, (2) crater, (3) tent, and (4) grass (Figure 1, Table 1, Supplemental Figures 1–6; frames G, I, J). All the unprocessed bulk polymer samples were flat and featureless with no protrusions or defects (Supplemental Figures 1–6, Frame G). Of the processed samples, only the anisotropic etched polycarbonate (aPC) was featureless Four polymeric surfaces, isotropic etched PolyImide (iPI), anisotropic etched Polyimide (aPI), anisotropic etch perfluoroalkoxy alkane (aPFA), and anisotropic etched Polyethylene terephthalate (aPET) had a nanoscale popcorn morphology (Table 2, Figure 1B: arrow; Supplemental Figures 1, 2).

**Table 2:** Morphologies of Polymeric Nanostructured Materials Generated by Plasma Etching

material/process	bulk	isotropic etched	anisotropic etched
polycarbonate (PC)	flat	crater	flat
polyimide (PI)	flat	popcorn	popcorn
perfluoroalkoxy alkane (PFA)	flat	tent	popcorn
polyethylene (PE)	flat	crater	popcorn
acrylonitrile butadiene styrene (ABS)	flat	crater	crater
acetal polyoxymethylene (POM)	flat	popcorn	popcorn
polyethyleneterephhalate (PET)	flat	grass	popcorn

The irregular, waffle-edged but evenly distributed popcorn structures ranged in size between 22 and 70 nm. The densities on the surfaces averaged between a minimum 10 features/1 μm^2^ for (which surface) to a maximum 100 of features/1 μm^2^ on the aPI and aPOM (Supplemental Figures 1, 2). Changes in the surface roughness of the bulk and etched surfaces were also determined using AFM (Table 3). In all but one case, the surface roughness increased after plasma etching. PET surfaces showed a reduction in roughness after an isotropic etch, which also displayed a “grass” surface topography with features that are fine and difficult to measure using the AFM.

**Table 3:** Surface Roughness (*S*_a_) and Root Mean Square (*S*_q_) of Samples Tested in This Paper

polymer	*S*_a_ (nm)	S_q_ (nm)	change compared to NT
**ABS**			
• NT	2.28	2.93	-
• 1D	4.58	6.93	increase
• 2D	10.40	13.68	increase
**acetal**			
• NT	2.59	3.38	-
• 1D	8.98	11.40	increase
• 2D	4.69	5.93	increase
**PI**			
• NT	0.81	1.03	-
• 1D	1.29	1.61	increase
• 2D	1.38	1.88	increase
**PC**			
• NT	0.21	0.43	-
• 1D	0.93	1.26	slight increase
• 2D	0.76	0.92	slight increase
**PET**			
• NT	1.04	1.34	-
• 1D	2.08	2.60	increase
• 2D	1.75	1.27	increase
**PFA**			
• NT	1.41	1.88	-
• 1D	2.70	3.45	increase
• 2D	3.24	4.17	increase
**PE**			
• NT	7.62	10.82	-
• 1D	6.28	8.08	decrease
• 2D	7.65	9.75	decrease

Four surfaces, isotropic etched polycarbonate (iPC), acrylonitrile butadiene styrene iABS, aABS, and isotropic etched polyethylene (iPE) had a crater surface morphology, which appear as irregular circles created because of the etching process (Table 2). The density of the craters on the surface of iPC was 5–10 per 10 μm^2^ with each crater between 100 and 400 nm in diameter. The craters on the iABS were at a lower density of only 2–3 per 10 μm^2^ but larger, between 300 and 500 nm in diameter (Figure 1c). The craters generated on the aABS surface were dense and interconnected. The tent configuration, which was present only on the iPFA, exhibited small pyramidal formations (between 200 and 400 nm) capped with 3–8 spherical structures that ranged in size between 25 and 50 nm in diameter. We only observed the “grass” morphology on a single etched surface, iPOM. The “grass” surface was composed of an interconnecting web of nanoscale thin tubes (∼25 nm in diameter) on ridged mounds (Supplementary Figure 2, second column, second row). Like many nature-inspired nanostructured surfaces, several of the fabricated surfaces also displayed hierarchical structures in the micron scale range.^21−23^ For instance, on iPFA, the surface had a popcorn morphology at the micron level. The surface of the iPE had parallel striations, while aPE exhibited preferential directional etching, with deep micrometer scale grooves that were composed of a popcorn texture.

### Bacterial Adhesion to Polymeric NSS Materials

**Figure 2:**
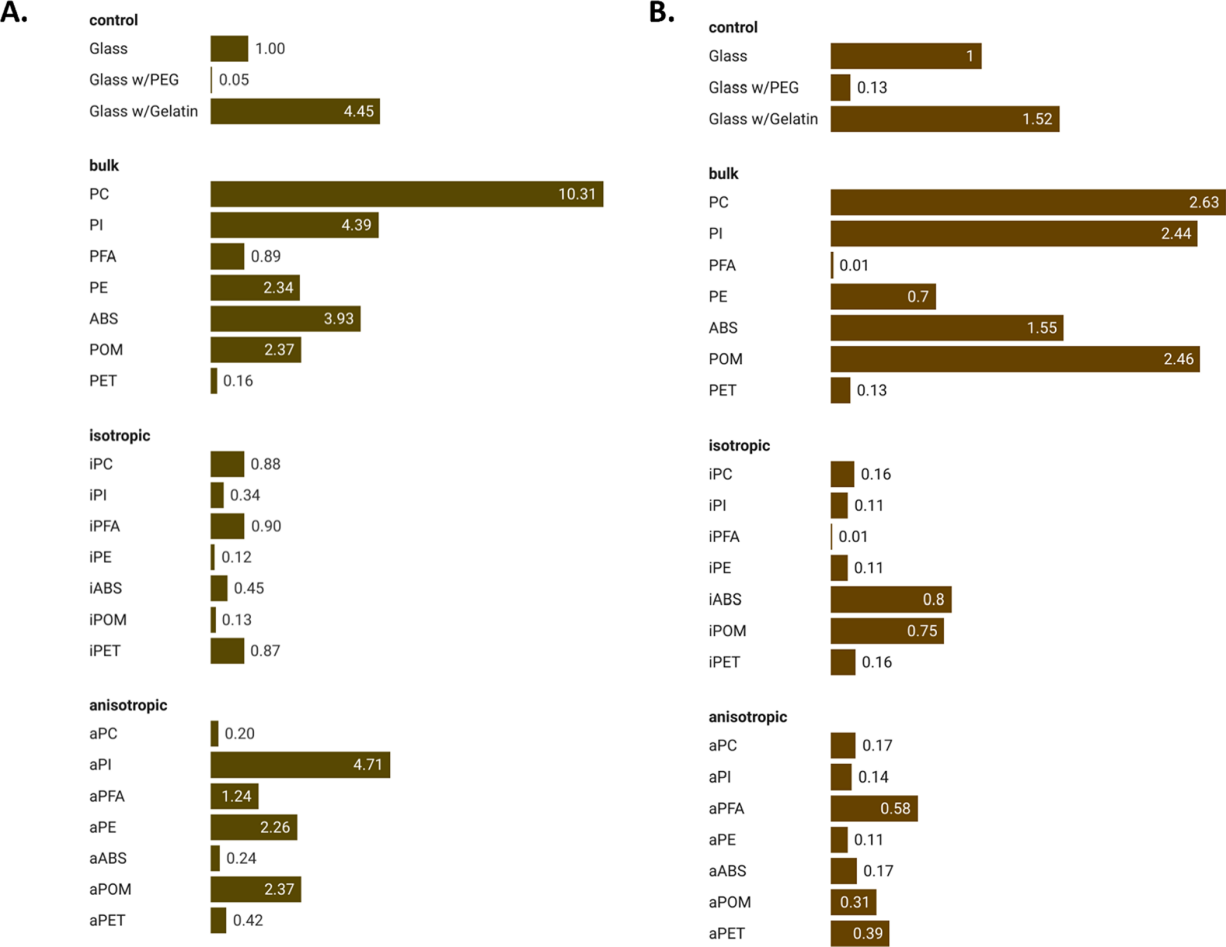
Fold difference of Cell/FOV on polymeric substrates relative to the untreated glass control. (A) After 1 h of contact with the surfaces; (B) after 24 h of contact.

Bacterial adhesion was determined using a GFP expressing *E. coli* cell line and a wash assay on the polymeric surfaces.^24^ As a control to establish a baseline for bacterial adhesion, we examined bacteria adsorption on untreated glass coverslips.^25^ As a negative control, we used polyethylene glycol (PEG)-treated glass slides – PEG treatment reduces cellular adhesive interactions with surface substrates. For additional positive controls, we also used gelatin-treated glass slides as gelatin treatment has been demonstrated to enhance the adhesion of several different cell types to surface substrates, including bacterial cells. As expected, we observed virtually no bacteria per field of view (10 μm^2^) on PEG-treated surfaces (2 ± 0.5 cells/10 μm^2^; Table 4; Figure 2) while glass surfaces treated with gelatin exhibited higher densities of bacterial cells when compared to untreated glass (175 ± 3 cells vs 39.67 ± 2.7 cells; Table 4; Figure 2).

**Table 4:** *E. coli* Adhesion to the Polymeric Surfaces Used in This Study^a^

surface	# cells/FOV	# cells/FOV
	1 h	24 h
glass	40 ± 3	537 ± 90
glass w/PEG	2 ± 0.5**	71 ± 15*
glass w/gelatin	177 ± 3**	815 ± 25*
PC	409 ± 90*	1411 ± 149**
iPC	35 ± 2	85 ± 2*
aPC	8 ± 1**	90 ± 14*
PI	174 ± 1**	1311 ± 114
iPI	14 ± 1**	61 ± 2*
aPI	187 ± 6**	75 ± 5*
PFA	35 ± 22	8 ± 3*
iPFA	36 ± 26	5 ± 1*
aPFA	49 ± 8	309 ± 5
PE	93 ± 54	374 ± 51
iPE	5 ± 1**	60 ± 3*
aPE	90 ± 3**	60 ± 4*
ABS	156 ± 2**	831 ± 39*
iABS	18 ± 10*	430 ± 8
aABS	10 ± 1**	93 ± 4*
POM	94 ± 2**	1320 ± 216*
iPOM	5 ± 2**	404 ± 9
aPOM	94 ± 5**	165 ± 3*
PET	6 ± 2**	70 ± 27*
iPET	35 ± 1	88 ± 4*
aPET	17 ± 2**	211 ± 42*

a *p* values for comparison between glass control and surfaces: * < 0.05, ** < 0.001.

Bacterial adhesion is influenced by surface morphology and other properties, and we predicted that changing the nanoscale surface texture of a polymeric material alters bacterial adhesion to these surfaces. We observed a wide range of bacterial adhesion levels to the nanostructured polymeric surfaces; however, the alteration to surface roughness did not play the predominant role in governing the interactions between the *E. coli* bacteria and the surfaces. Two materials, PFA and PET, did not exhibit any differences in adhesion of the bacteria between the bulk and etched counterparts after one hour of contact (Table 4, Figure 2), although these surfaces displayed significant changes in surface architecture (Table 1). Other etched polymer materials, including PC, PI, PE, ABS, and POM materials, showed a significant increase in the number of bacteria/per unit area when compared to the bulk (Table 4, Figure 2). In other cases, etching of the polymeric surfaces lowers bacterial cell adhesion. For instance, we observe the highest level of bacterial cell adhesion on the flat unprocessed bulk PC when compared to isotropic- and anisotropic-etched PC surfaces, while bacterial adhesion of plasma etched PC surfaces was significantly reduced, including isotropic and anisotropic etched PC surfaces, both of which have a significant surface structure (Table 1).

Several polymeric substrates demonstrated reduced affinity to bacteria cells when compared to the glass coverslip control, although none were like the PEG-glass negative control. These surfaces included the only surfaces to have fewer number of cells: iPC, iPI, iPE, iABS, aABS, iPOM, bPET, and aPET (Table 4). The common trend of each of these surfaces is that they tend to be hydrophilic.

We also examined bacterial adhesion after 24 h incubation on these surfaces. In all but four cases, we observed an increase in the number of cells on the surface when compared to a 1 h incubation (Supplemental Figure 8). However, within the material, i.e., comparing PE versus iPE or aPE, we observe less of a difference in the number of cells per unit area between surfaces, which suggests that bacteria have the capacity to overcome surfaces with properties that were not initially optimal for colonization. In four cases (bPFA, iPFA, aPI, and aPE; Supplementary Figures 2F, 3B, D, and 7F), we observed fewer cells/0.1 mm^2^ at 24 h than at 1 h, which suggests that the processing of these materials inhibit the growth of bacterial biofilms. These results suggest that there is no relationship between the surface energy/hydrophobicity and *E. coli* adhesion nor do we observe a relationship between a specific type of nanoscale surface feature and cell adhesion. However, some polymeric materials are inherently more adhesive than others to *E. coli* cells, demonstrating that surface composition predominates the interaction between the cell and the surface.

### Assessment of Bacterial Membrane Integrity

**Figure 3:**
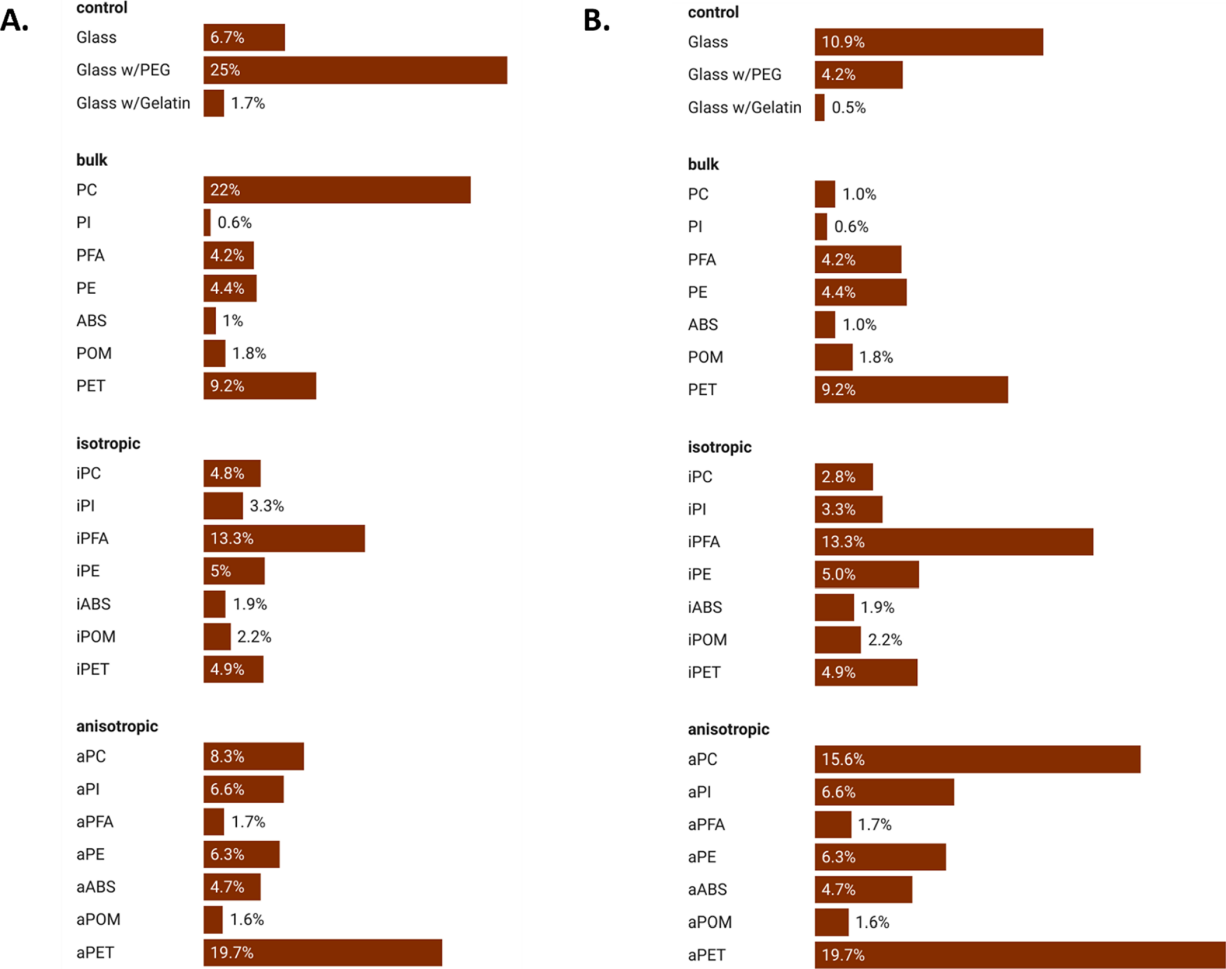
Percentage of PI-labeled *E. coli* cells exhibiting a loss of plasma membrane integrity in contact with polymer surfaces. (A) After 1 h of contact with the etched polymeric surfaces; (B) after 24 h of contact.

**Figure 4:**
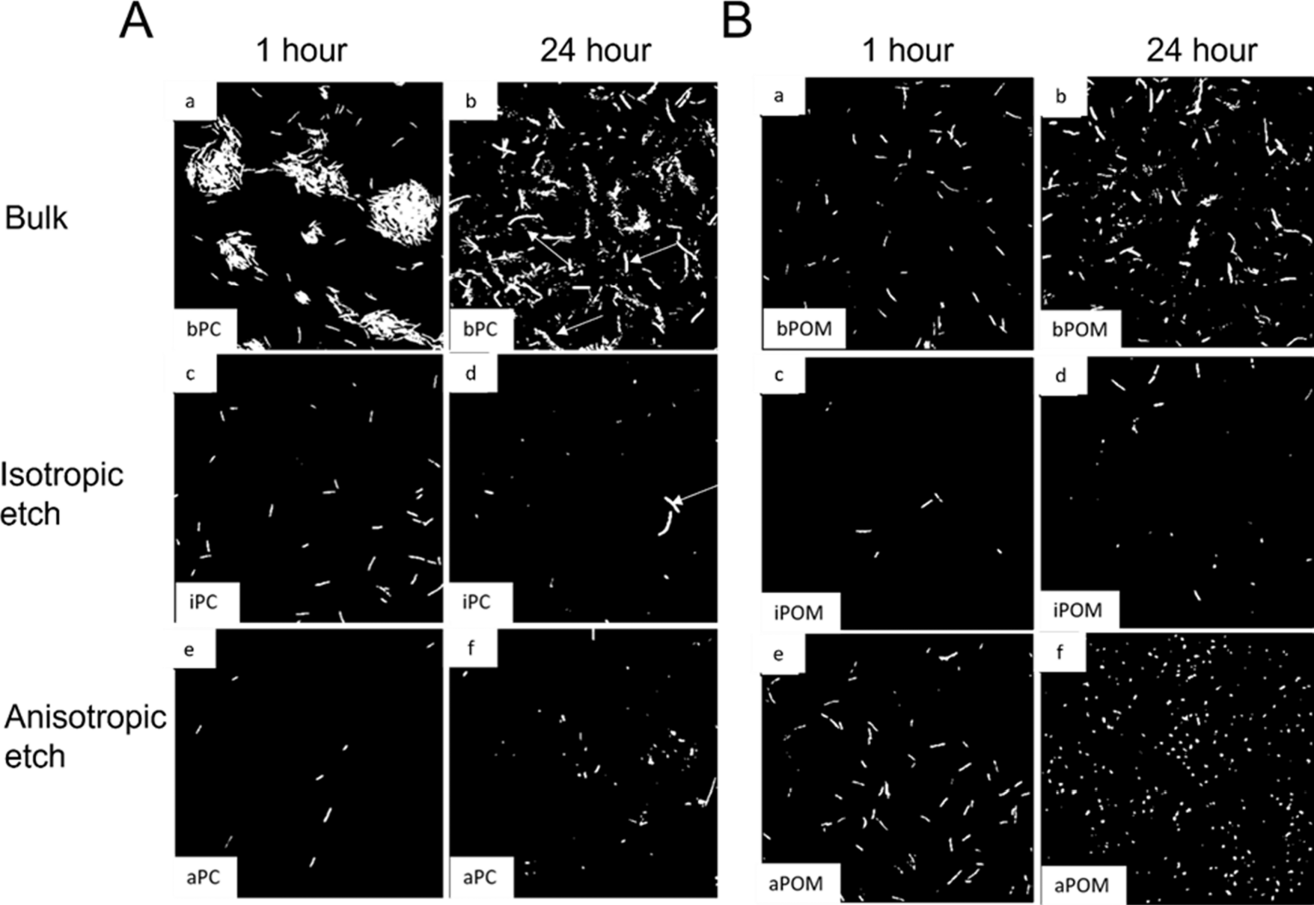
Examples of changes to *E. coli* initial colonization behavior and morphology as a response to plasma-etched polymer surfaces, specifically. All bacterial cells have been labeled with GFP expression and viewed under a confocal microscope. (A) Polycarbonate (PC) and (B) acetal polyoxymethylene (POM) materials. A i, ii: colonization of a bulk PC surface by *E. coli* bacteria; A I, the initial colonization events after 1 h incubation show that those bacteria interact with the PC in clumps of cells; A ii, after 24 h, the bacteria spread across the surface diffusely and have an elongated morphology. A iii, iv: colonization of an isotopic etched PC surface by *E. coli* bacteria. A iii, the initial colonization of bacteria is diffuse, and bacteria show a normal morphology; A iv, few bacteria occupy the surface after 24 h and the bacterial cells appear smaller in length, although some cells are elongated. A v, vi: colonization of an anisotropic etched PC surface by *E. coli* bacteria. A v, a few small, dispersed bacteria adhere to the surface after 1 h incubation; A vi, after 24 h, many of the cells are small and of different shapes and sizes. B i, ii: colonization of a bulk POM surface by *E. coli* bacteria. B i, bacteria are initially distributed across the POM and exhibit normal cell shape and morphology; B ii, after 24 h, more cells are dispersed across the surface and show a range of sizes and shapes, including small and circular. B iii, iv: colonization of an isotopically etched POM e surface by *E. coli* bacteria. B iii, the initial colonization of bacteria is diffuse with only a few bacteria that show a normal morphology; B iv, fewer bacteria occupy the surface after 24 h and the bacterial cells appear smaller. B v, vi: colonization of an anisotropic etched polycarbonate surface by *E. coli* bacteria. B v, after 1 h incubation, the surface has been colonized by normal sized and shaped bacteria that are evenly dispersed across; B vi, after 24 h, many small *E. coli* cells are dispersed across the surface.

Microbial interactions with many chemicals and materials, including many nanoscale materials, disrupts the plasma members, which often results in a reduction in viability.^26^ To determine whether interactions of *E. coli* with our nanostructured polymeric surfaces occur, we examined the changes to the permeability of the bacterial plasma membrane, using the vital dye propidium iodide (PI). PI is a nucleic acid dye that is plasma membrane impermeable and will only label cells that have a disrupted plasma membrane.^26^ Bacterial cells exposed to untreated glass substrates exhibited a background level of 6.72 ± 0.27% of PI-labeled cells. In the positive control experiment of sodium-hypochlorite-treated bacterial cells, we observed 100% of the cells labeling with PI. *E* on our experimental surfaces exhibited a range of cells exhibiting plasma membrane perturbation. Some surfaces resulted in an elevated level of plasma membrane disruption as demonstrated by PI labeling: bPFA, 63.21 ± 3.78%, iPFA 63.55 ± 4.77%, bPE 58.06 ± 1.41% and iABS 54.72 ± 2.80% all exhibited high percentage of *E. coli* cells that labeled with PI after 1 h of contact, suggesting that these surfaces may damage or stress the integrity of the plasma membrane (Figure 3). However, other surfaces, even those with the same composition as the plasma membrane disrupting surfaces, showed no significant PI labeling when compared to that of the controls (Figure 4). For example, the percentage of permeable cells on the following surfaces, iPC, aPC, all the PI substrates, aPE, bABS, bPOM, aPOM, iPET, and aPET, was within the range we had accounted for using the glass coverslip control substrate. In many cases, there were significant differences in the percentage of PI-labeled cells on substrates that are composed of the same material but have different surface topography due to the etching process, suggesting that plasma membrane stress may be the result of bacterial interaction with specific features of these surfaces. However, as with adhesion, there was no correlation between the general classification of surface features or surface composition that predicts plasma membrane perturbation. The only material to exhibit a significant difference in PI-labeled *E. coli* when compared to the substrate control (∼10%) after 24 h exposure was the popcorn surfaced aPET, in which we observed a higher number of membrane perturbed cells, aPET at a percentage of 19.67 ± 0.27%). In many cases, PI-labeled cells are inviable or dying; however, there has been some evidence that microbes may tolerate higher levels of membrane permeability and thus label with PI but not be dead or dying.^27^

### Changes to the Bacterial Morphology

Stress often alters the bacterial morphology,^28,29^ and we also observed changes to the bacterial cell morphology and colonializing behavior on surface-modified polymeric materials including changes in the cell length, the presence of additional cellular appendages, and in several cases, changes to the bacteria’s initial surface colonizing behavior after 1 h of incubation (Table 5). On untreated glass surfaces *E. coli* bacteria have a cylindrical shape that is 3–5 μm long. We observe changes in the length of the bacteria when incubated on different materials. Several surfaces, including iPC, bPFA, bPET, and aPET (Table 5, Figure 4, Supplemental Figures 5, 7) trigger the formation of shorter bacteria that are less than 3 μm and appear as small round cells; the smaller cell phenotype is more prominent after 24-h incubation. In some cases, the cells themselves appear small and irregular in shape; however, cells incubated on aPOM surfaces for 24 h have a uniform small round phenotype (Figure 4Bf, suggesting that some aspect of this surface, perhaps its articulated “popcorn” nanoscale features are inducing this shape and size phenotype). A few surfaces do the opposite and induce the formation of elongated bacterial cells, which are greater than ≥10 μm in length (Figure 4Ae, arrows). In addition to shape and size changes, we also found that *E. coli* bacteria colonize the surfaces differently. In most cases, the bacteria deposit onto a surface in a uniform dispersion; however, in a few cases, such as with the bulk PC surface, we observe an initial clumping behavior of bacteria (Figure 4Aa).

**Table 5:** Summary of Bacterial Morphological Changes to Polymer Surfaces

material/etching process	bulk		isotropic etched		anisotropic etched	
	colonization behavior/shape		colonization behavior/shape		colonization behavior/shape	
	1 h	24 h	1 h	24 h	1 h	24 h
porycarbonate (PC)	clustered/normal	clustered/elongated	dispersed/normal	dispersed/normal	clustered/normal	dispersed/normal
polyimide (PO)	dispersed/elongated	dispersed/normal	dispersed/normal	dispersed/normal	dispersed/normal	dispersed/normal
perfluoroalkoxyalkane (PFA)	dispersed/small	dispersed/normal	dispersed/normal	dispersed/normal	dispersed/normal	dispersed/normal
polyethylene (PE)	clustered/normal	dispersed/normal	dispersed/normal	dispersed/small	dispersed/normal	dispersed/small
acrylonitrile butadiene styrene (ABS)	dispersed/small	dispersed/normal	dispersed/normal	dispersed/normal	dispersed/normal	dispersed/small
acetal polyoxymethylene (P0M)	dispersed/small	dispersed/normal	dispersed/normal	dispersed/small	dispersed/normal	dispersed/small
polyethylene terephalate (PET)	dispersed/small	dispersed/normal	dispersed/small	dispersed/small	dispersed/small	clustered/small

**Figure 5:**
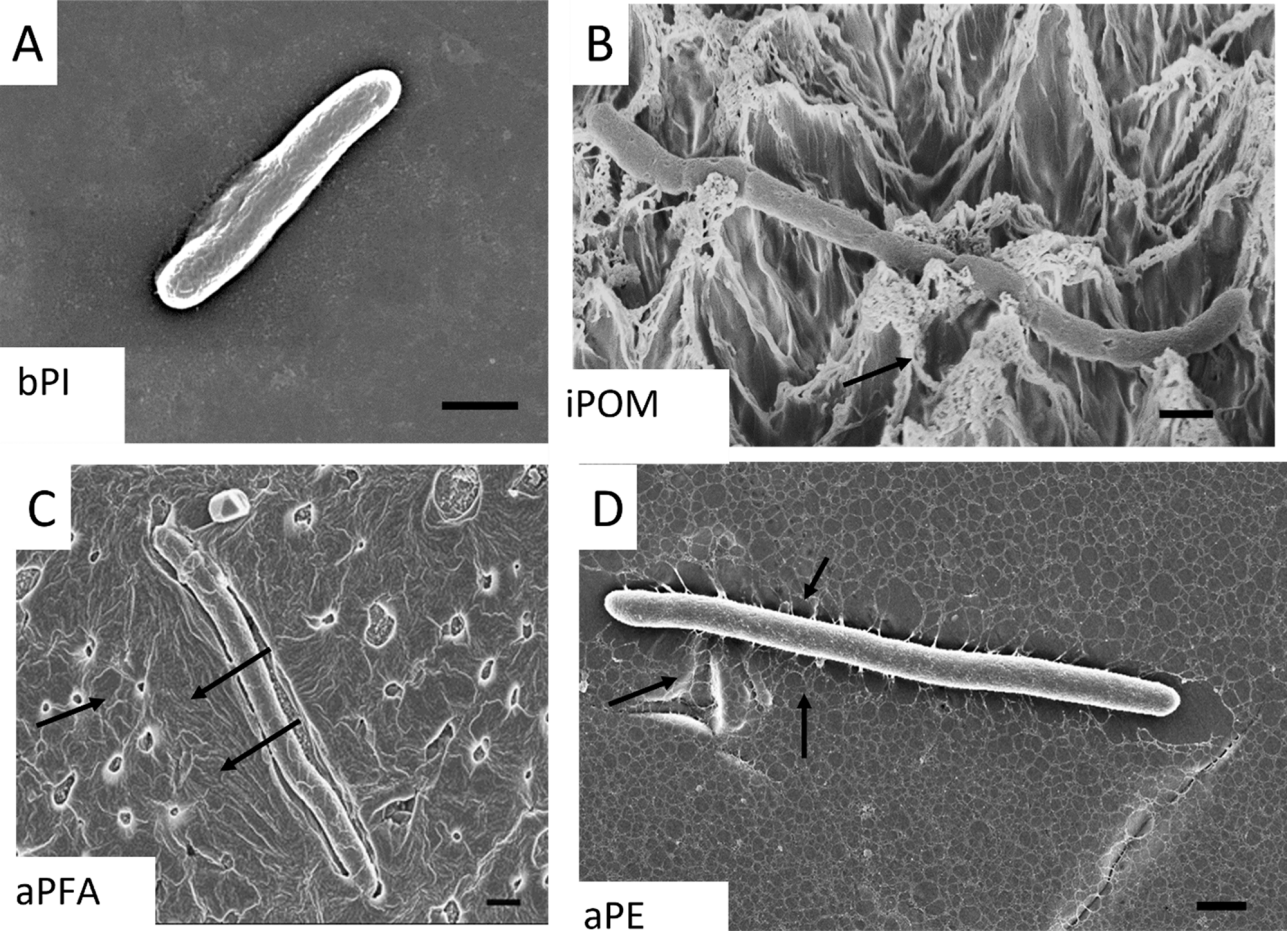
Changes to *E. coli* extracellular materials when in contact with plasma etched polymer surfaces. (A) *E. coli* cell on a glass coverslip, showing cylindrical morphology and minimal extracellular material, scale bar, 1 μm; (B) *E. coli* cell in the rough isotopically etched POM “popcorn” surface has an elongated morphology and many fine nanoscale projections attached to the surface features (arrows), scale bar; (C) *E. coli* bacterium on anisotropic etched PFA “popcorn” surface showing the deposition of extracellular materials that appear as high contrast edges around the elongated cell (arrows) as well as some of the surface features, scale bar is 1 μm; (D) elongated *E. coli* bacterium on anisotropic etched PE “popcorn” surface exhibiting many extensions with the surface structures which appear to be part of the surface as well as the cell (arrows), scale bar 1 μm.

In addition to these morphological differences, we also observed qualitative differences in the presence of different cellular projections in cells associated with different surfaces (Figure 5). On most surfaces, the bacteria exhibit a cylindrical morphology (Figure 5a) which includes the presence of bacterial surface adhesion appendages. The bacteria on iPOM expressed increased surface area to maximize contact points (Figure 5b). *E. coli* on aPFA and aPC appear to secrete materials that bridge contact points on the surface (Figure 5 c,d) reaching out for adhesion points on the surfaces.

## Discussion

Preventing the initial adhesion of bacteria is a key step in controlling biofilm formation, but as demonstrated here, the bacterium *E. coli* assumes multiple morphologies in response to different surfaces and possibly has different strategies for colonizing surfaces. In this study, we have shown that the plasma surface modification of polymeric materials alters *E. coli* cell adhesion, viability, cell morphology, and certain cell behaviors such as the initial surface colonization. The diverse behavior displayed by *E. coli* in response to a variety of different surfaces suggests that specific material properties or combinations of surface properties may trigger different colonization mechanisms. As presented here, plasma etching generated a uniform reduction in bacterial cell adhesion in all surfaces and increased general submicro/nanoscale surface roughness; however, the resulting changes to cell behavior did not follow any trend in the qualitative surface structure or surface energy. High-aspect-ratio nanoscale structures alter cell viability through mechanical interrogation of the cell;^30−34^ however, lower aspect ratio structures trigger different responses. Submicron scale features on a surface inhibit adhesion and colonization;^8^ our work in this study supports these findings. While nanoscale surface textures may control some cell behaviors, such as adhesion or morphology, we observed no relationship between similar surface architectures, changes in surface roughness, and behavior, suggesting that something other than these properties plays to the bacterium response. Recent work that examined the role that surface charge plays in bacterial adhesion concluded that the physical properties of a surface are not critical for bacteria/surface adhesion.^9^ This, along with our findings, suggests that the bacteria are more complex and responsive to different surface contexts. These results strongly suggest that a yet unknown bacterial mechanism or set of mechanisms controls these interactions. Our results suggest that *E. coli* biofilm formation and behavior involves one or more factors or combinations of factors that include surface composition. Predicting the outcome of an interaction between a bacterium and a surface is complicated by this complexity, a combination of chemical, physical, and mechanical properties as well as interactions with components not directly associated with the bacterial cell.

In this study, we observed several different responses of *E. coli* bacteria when colonizing abiotic surfaces such as the clustering of colonizing bacteria on bulk PC surfaces which suggests something about this surface triggers and promotes cluster aggregation, perhaps acting under some sort of cooperative adhesion. In the case of plasma-treated PC, this aggregation-inducing property is lost. In other examples, we observed that the immediate high levels of cell permeabilization/death associated with specific materials such as PFA or ABS materials were overcome by the bacteria after 24 h resulting in robust biofilm. In other cases, such as PET materials, the cell never recovered. These results suggest that immediate cell death from contact may not be a reasonable indication of antimicrobial activity, and in fact, may be part of a natural response of a clonal microbial organism to a noxious substrate.

The bacteria/surface interaction is complicated by specific bacterial responses that are pertinent for biofilm formation including communal response via quorum sensing.^10^ More work needs to explore the bacterial responses to a variety of surfaces. In the context of high aspect ratio antimicrobial surfaces, this has been limited to measuring the loss of viability of a bacterium or other microbes when they encounter a material, but other materials may be changing aspects of the microbe communities, such as metabolism and the production of secondary analytes that not only control a microbe’s behavior but also the behavior of the local microbiome.^35,36^ Bacteria and other microbes have evolved responses to form biofilm on countless substrates and under countless environmental challenges, and clonal organisms require only a single cell to survive any encounter to maintain their existence.^37−39^ Future work needs to focus on identifying these mechanisms and correlating their requirements for specific surface properties whether they are chemical, mechanical, or structural.

## Supporting Information Available

Collage of representative images for contact angle analysis and representative SEM micrographs of surfaces used in these experiments; collages containing representative confocal Light micrographs of 1 and 24 h *E. coli*; biofilms on the polymeric surfaces that depict surface colonization; and SEM images of morphological changes to bacterium attached to surfaces (PDF)
