# Supplementary material for: Escherichia coli Adhesion and Biofilm Formation on Polymeric Nanostructured Surfaces
Source: ACS Omega. 2023 Oct 9;8(50):47520–9. doi: 10.1021/acsomega.3c04747 (PMC10734028; doi:10.1021/acsomega.3c04747)

Supplemental Materials

Title: *Escherichia coli* adhesion and biofilm formation on polymeric nanostructured surfaces

Authors: Divya Iyer, Eric Laws, and Dennis LaJeunesse*

Address: Department of Nanoscience, Joint School of Nanoscience and Nanoengineering, University of North Carolina Greensboro, 2907 East Lee Street, Greensboro, North Carolina, 27455

Supplemental Figure 1: SEM and Contact angle (insets) analysis of PE, PI and PFA surface materials. Top row Bulk (cleaned but unprocessed materials), Middle row materials processed via a dry O2 Plasma isotopic etch, Bottom row Materials processed via an anisotopic etch. All scale bars 1um.

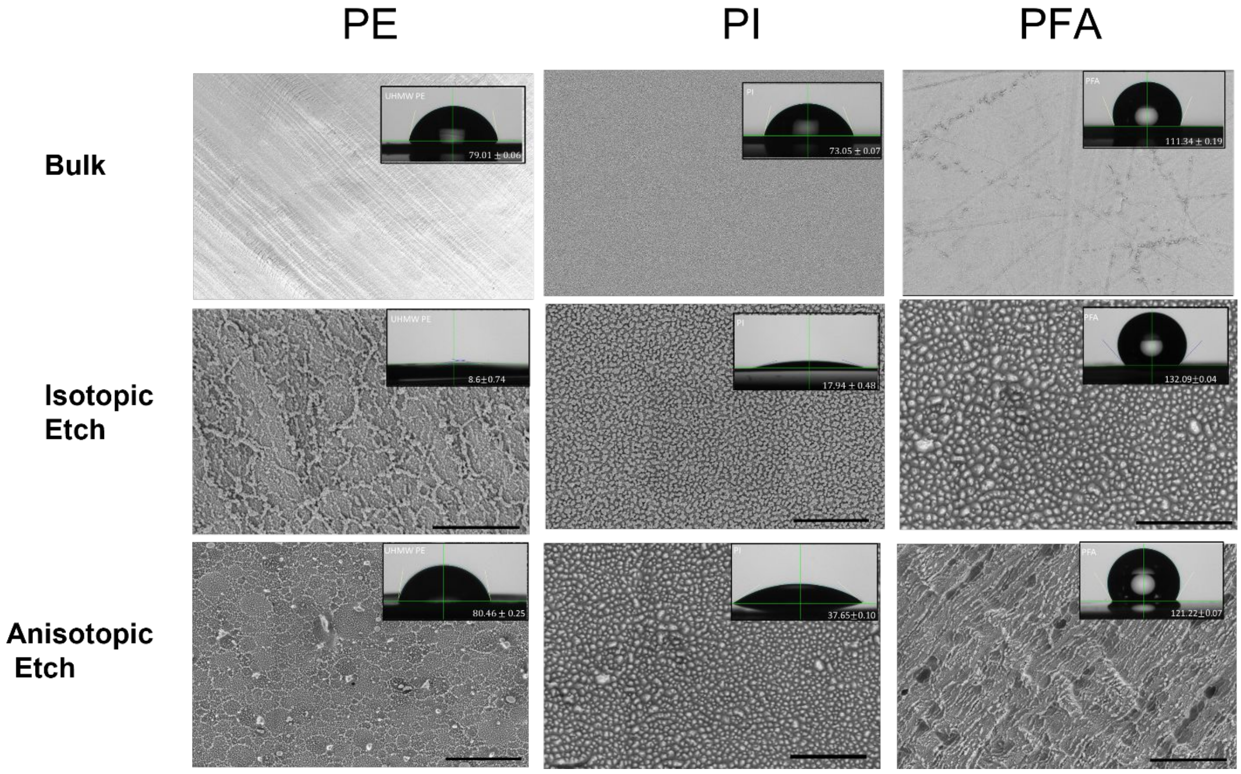

Supplemental Figure 2: SEM and Contact angle (insets) analysis of PET, POM, and ABS surface materials. Top row Bulk (cleaned but unprocessed materials), Middle row materials processed via a dry O2 Plasma isotopic etch, Bottom row Materials processed via an anisotopic etch. All scale bars 1um.

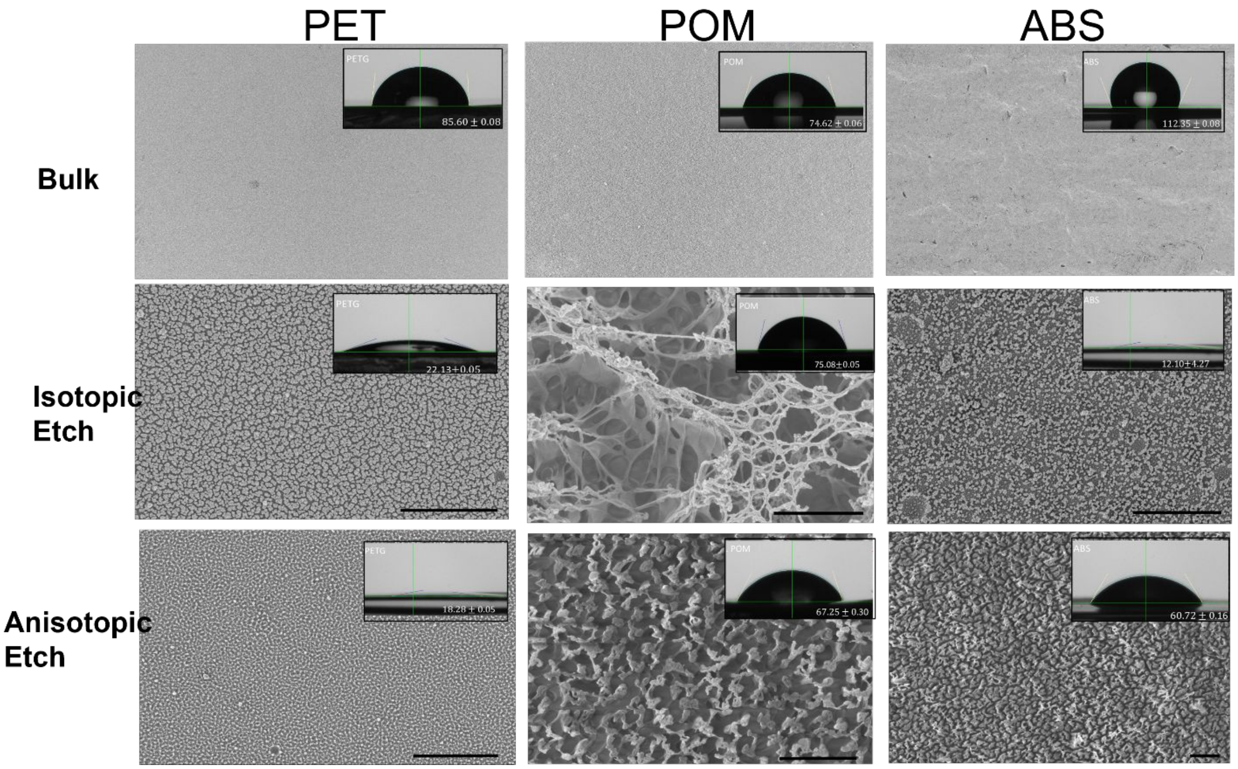

Supplemental Figure 3. Changes *E. coli* colonization behavior and morphology when in contact with plasma etched polymer surfaces with PE materials. First column after a 1 hour exposure, second column after a 24 hour exposure; third column SEM micrographs of a typical cells after one hour exposure. Top row Bulk (cleaned but unprocessed materials), Middle row materials processed via a dry O2 Plasma isotopic etch, Bottom row Materials processed via an anisotopic etch.

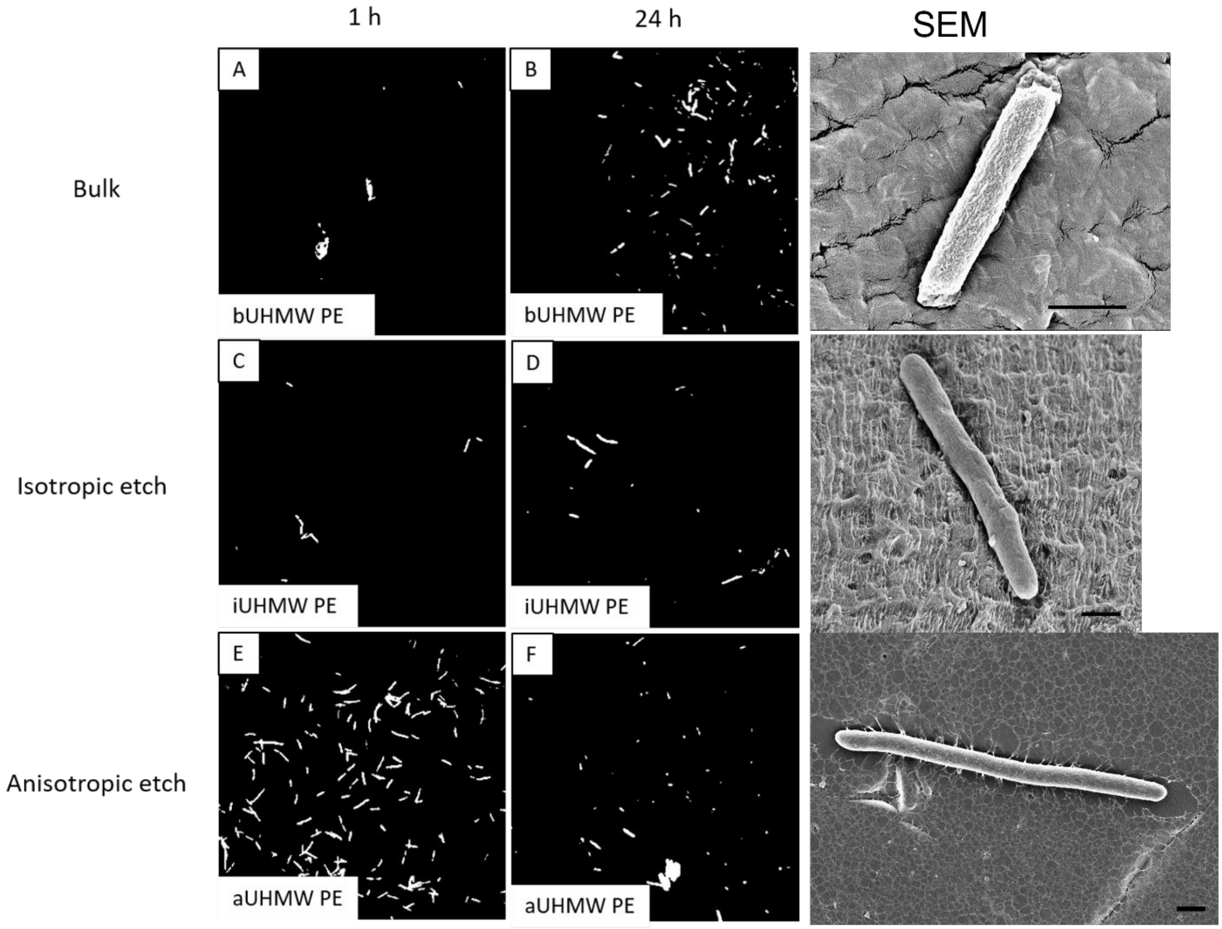

Supplemental Figure 4. Changes *E. coli* colonization behavior and morphology when in contact with plasma etched polymer surfaces with PI materials. First column after a 1 hour exposure, second column after a 24 hour exposure; third column SEM micrographs of a typical cells after one hour exposure. Top row Bulk (cleaned but unprocessed materials), Middle row materials processed via a dry O2 Plasma isotopic etch, Bottom row Materials processed via an anisotopic etch.

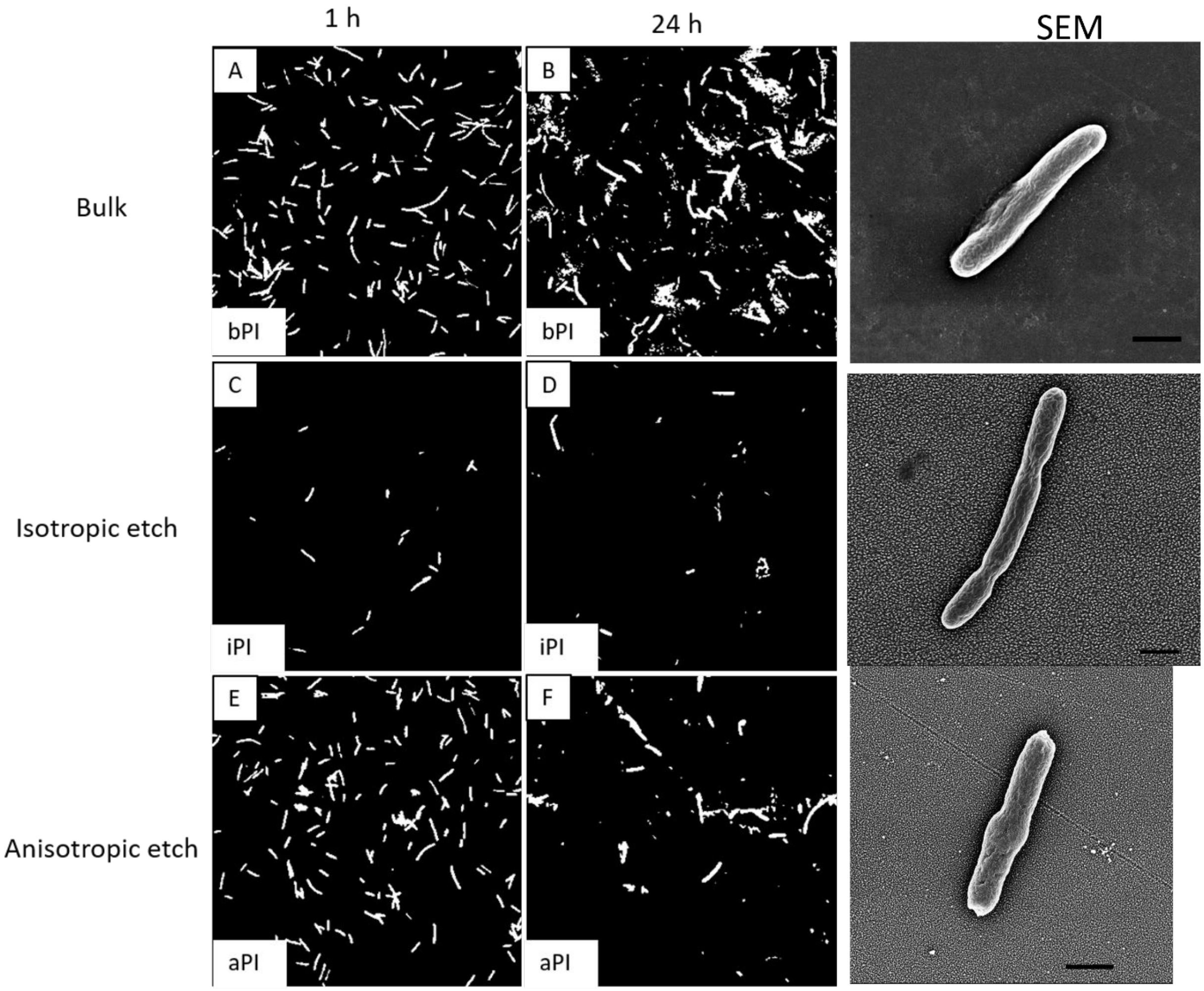

Supplemental Figure 5. Changes *E. coli* colonization behavior and morphology when in contact with plasma etched polymer surfaces with PFA materials. First column after a 1 hour exposure, second column after a 24 hour exposure; third column SEM micrographs of a typical cells after one hour exposure. Top row Bulk (cleaned but unprocessed materials), Middle row materials processed via a dry O2 Plasma isotopic etch, Bottom row Materials processed via an anisotopic etch.

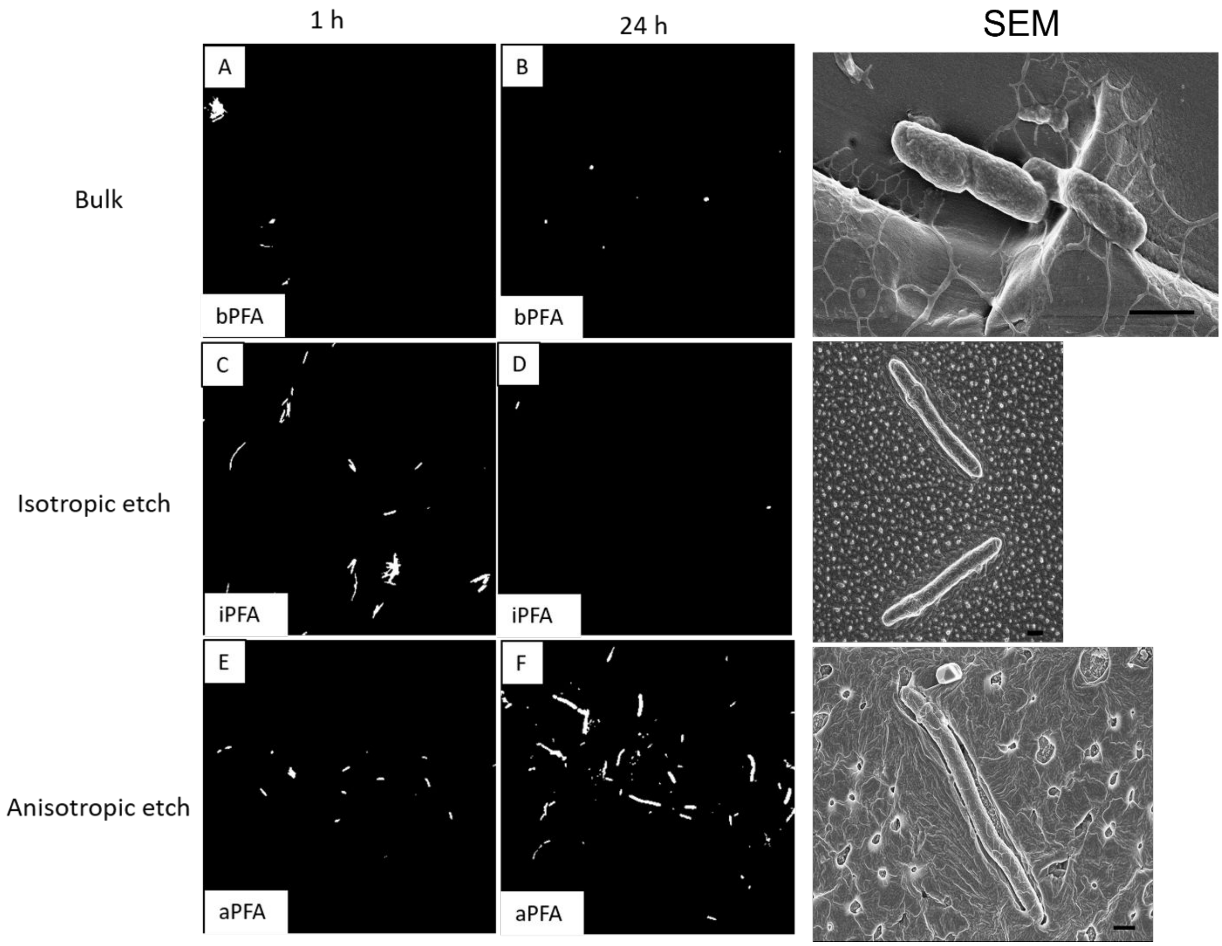

Supplemental Figure 6. Changes *E. coli* colonization behavior and morphology when in contact with plasma etched polymer surfaces with ABS materials. First column after a 1 hour exposure, second column after a 24 hour exposure; third column SEM micrographs of a typical cells after one hour exposure. Top row Bulk (cleaned but unprocessed materials), Middle row materials processed via a dry O2 Plasma isotopic etch, Bottom row Materials processed via an anisotopic etch.

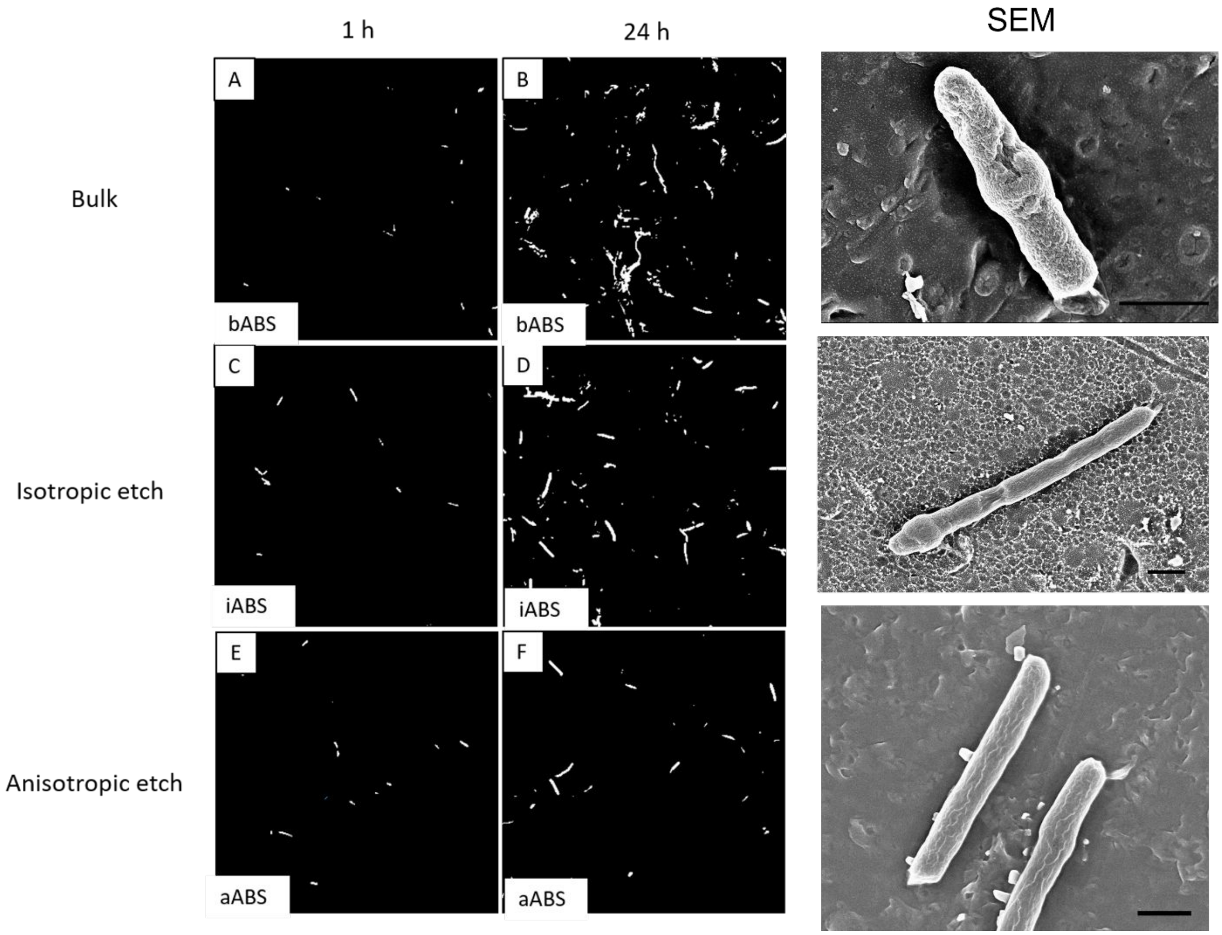

Supplemental Figure 7. Changes *E. coli* colonization behavior and morphology when in contact with plasma etched polymer surfaces with PET materials. First column after a 1 hour exposure, second column after a 24 hour exposure; third column SEM micrographs of a typical cells after one hour exposure. Top row Bulk (cleaned but unprocessed materials), Middle row materials processed via a dry O2 Plasma isotopic etch, Bottom row Materials processed via an anisotopic etch.

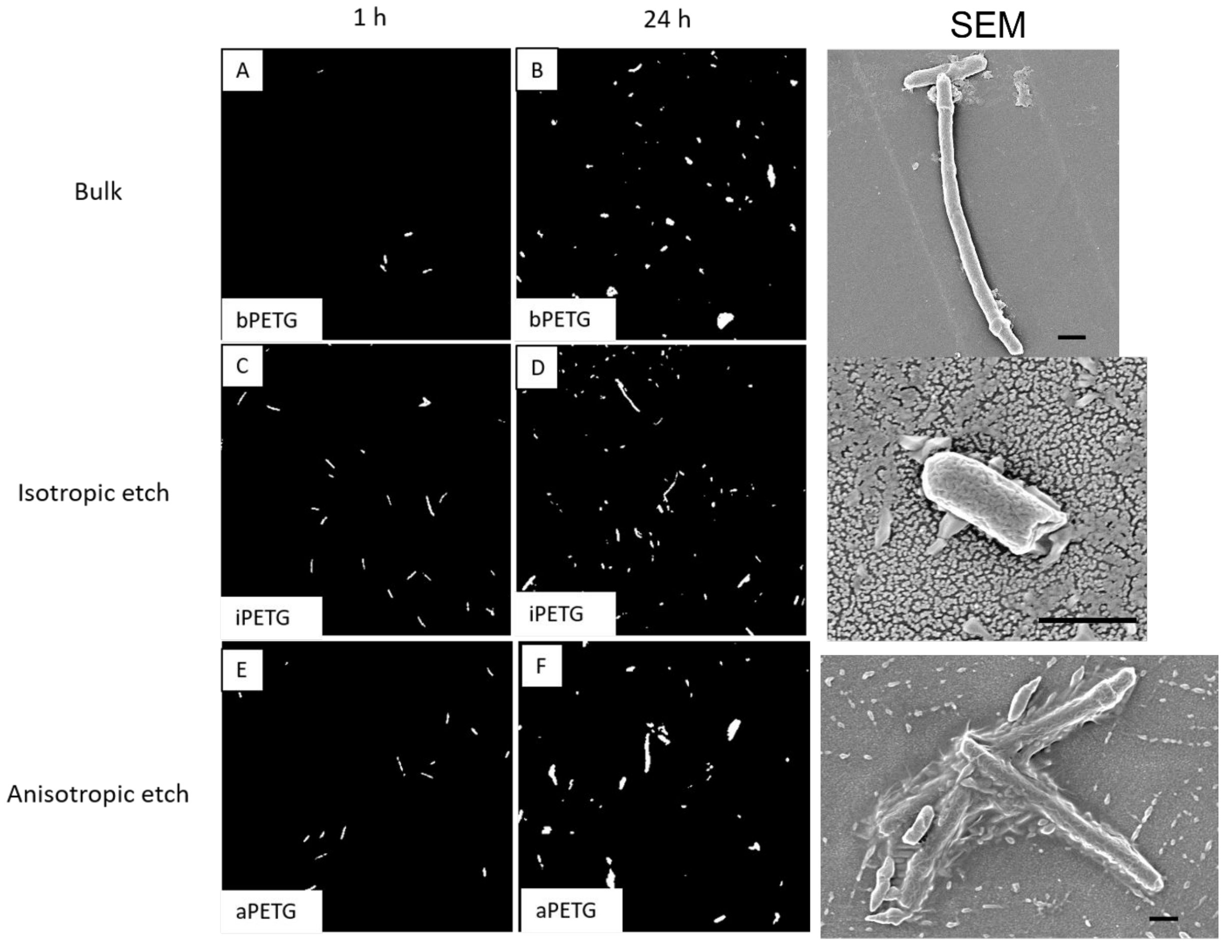

Supplemental Figure 8. Changes *E. coli* colonization behavior and morphology when in contact with plasma etched polymer surfaces with POM materials. First column after a 1 hour exposure, second column after a 24 hour exposure; third column SEM micrographs of a typical cells after one hour exposure. Top row Bulk (cleaned but unprocessed materials), Middle row materials processed via a dry O2 Plasma isotopic etch, Bottom row Materials processed via an anisotopic etch.

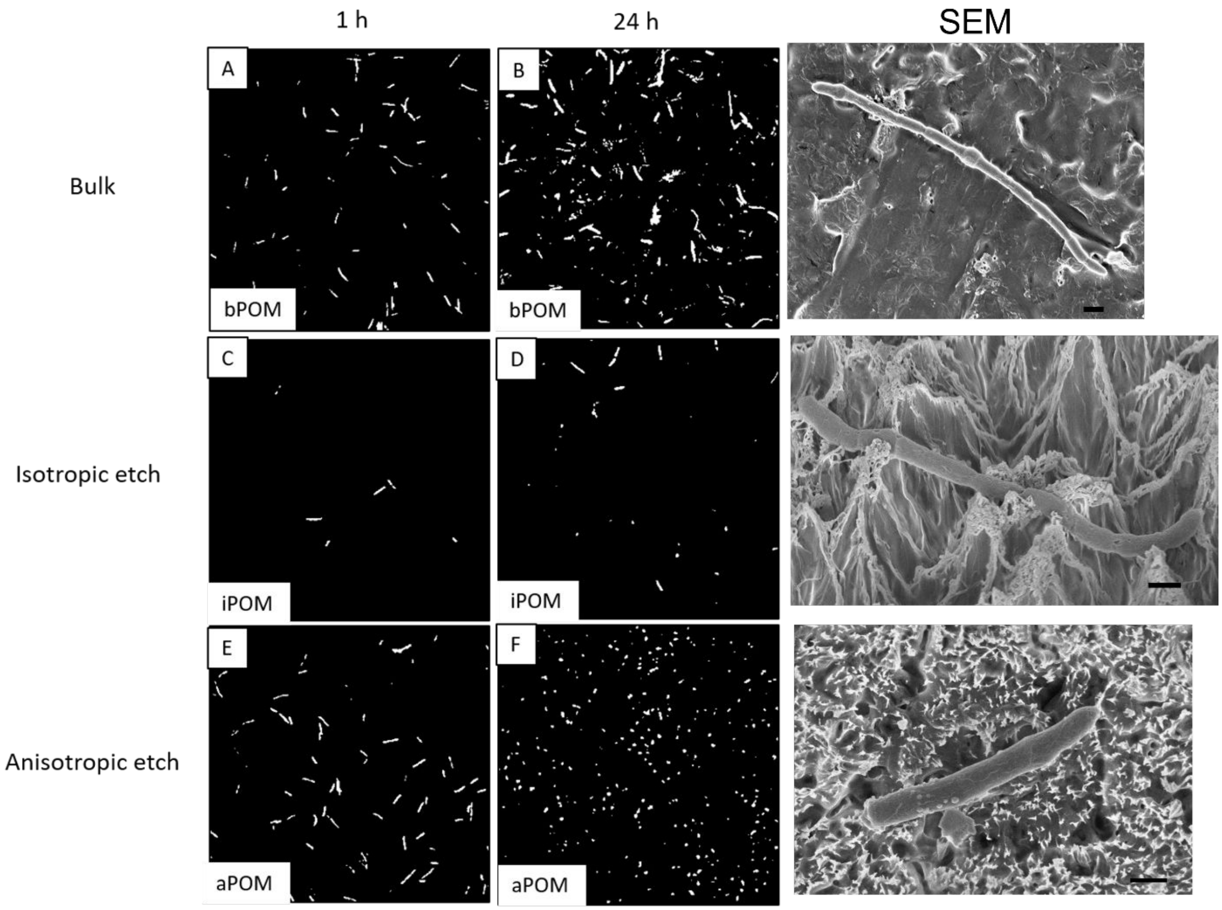

Supplement: Supplementary file 1 — ao3c04747_si_001.pdf [file ao3c04747_si_001.pdf]
